# No Detectable Effect of the DNA Methyltransferase DNMT2 on *Drosophila* Meiotic Recombination

**DOI:** 10.1534/g3.114.012393

**Published:** 2014-08-27

**Authors:** Caiti S. Smukowski Heil

**Affiliations:** Biology Department, Duke University, Durham, North Carolina 27708

**Keywords:** DNA methylation, DNMT2, recombination, *Drosophila*, epigenetics

## Abstract

Epigenetics is known to be involved in recombination initiation, but the effects of specific epigenetic marks like DNA methylation on recombination are relatively unknown. Studies in Arabidopsis and the fungus *Ascobolus immersus* suggest that DNA methylation may suppress recombination rates and/or alter its distribution across the genome; however, these patterns appear complex, and more direct inquiries are needed. Unlike other organisms, *Drosophila* only have one known DNA methyltransferase, DNMT2, which is expressed in the ovaries and historically has been thought to be responsible for limited genomic DNA methylation. To test for a role of DNMT2 on the frequency and distribution of recombination, I compared recombination rates between *Dnmt2* −/− and *Dnmt2* +/− *Drosophila melanogaster* individuals in two euchromatic regions and one heterochromatic region across the genome. I failed to detect an altered pattern of recombination rate in the absence of DNMT2 in all regions surveyed, and conclude that other epigenetic effects are regulating recombination initiation in *Drosophila*.

Epigenetics has long been predicted to play a role in the initiation of meiotic recombination. Observations of variation in recombination rate within and between individuals, sexes, populations, and across the genome (such as euchromatin *vs*. heterochromatin) suggest a role beyond DNA sequence in determining locations of recombination events (Lichten and Goldman 1995; Barthes *et al.* 2011). An association between open chromatin formation and double strand breaks, the first step in the initiation of recombination, has been identified in yeast, dog, and several plants (Berchowitz *et al.* 2009; Pan *et al.* 2011; Auton *et al.* 2013; Choi *et al.* 2013; Hellsten *et al.* 2013), and the histone H3K4 methyltransferase PRDM9 influences the distribution of recombination sites in human and mouse (Buard *et al.* 2009; Baudat *et al.* 2010; Berg *et al.* 2011; Grey *et al.* 2011; Brick *et al.* 2012; Acquaviva *et al.* 2013; Sommermeyer *et al.* 2013). However, the possible roles of specific epigenetic marks apart from H3K4me3 are less understood. For example, the relation between recombination initiation and the best-characterized epigenetic factor, DNA methylation, appears complex and remains relatively unexplored.

DNA methylation describes the transferring of a methyl group (CH_3_) to the 5th position of a cytosine residue, typically at CpG sites and repeat elements (Robertson and Wolffe 2000). The reaction is catalyzed by a family of conserved proteins known as DNA methyltransferases: DNMT1, the maintenance methyltransferase, ensures proper inheritance of methylation patterning after replication in somatic cells; DNMT3s (3A, 3B, 3L), the *de novo* methyltransferases, establish DNA methylation patterns during embryogenesis; and DNMT2, an enigmatic methyltransferase with conserved catalytic motifs, has a historically disputed function (Yoder *et al.* 1997; Dong *et al.* 2001; Schaefer and Lyko 2010; Barthes *et al.* 2011; Krauss and Reuter 2011). These genes function within an ancient regulatory mechanism shared by animals, plants, and fungi, serving in diverse roles often related to repression of gene expression (Feng *et al.* 2010; Zemach *et al.* 2010; Zemach and Zilberman 2010; Jurkowski and Jeltsch 2011; Nanty *et al.* 2011).

A link between DNA methylation and recombination was first hypothesized by Rossignol and Faugeron (1994) and Yoder *et al.* (1997) in which DNA methylation promotes genome integrity through the suppression of recombination between dispersed repetitive sequence. More direct evidence of a functional link between DNA methylation and recombination is somewhat limited, but a study in the fungus *Ascobolus immersus* showed crossover formation was reduced several hundred fold in an *in vivo* methylated hotspot compared with an unmethylated hotspot (Maloisel and Rossignol 1998). More recently, several reports in Arabidopsis paint a more nuanced pattern. For example, in the absence of MET1 (the DNMT1 homolog), researchers independently observed a pattern of increased recombination in euchromatin and decreased recombination in (typically hypermethylated, transposon rich) heterochromatin (Melamed-Bessudo and Levy 2012; Mirouze *et al.* 2012; Yelina *et al.* 2012). Each study found that the total number of crossover events was not different between *met1* mutants and the wild type, indicating that the loss of DNA methylation affects the distribution of crossovers but not their overall number. When wild-type Arabidopsis transcription start and termination sites are examined specifically, DNA methylation is decreased in recombination hotspots relative to transcription start or termination sites in which recombination was absent (Choi *et al.* 2013), supporting data from *A. immersus*. In contrast, some indirect evidence in humans pointed to a positive association between recombination rate and DNA methylation (Sigurdsson *et al.* 2009).

In this study, I explore the complex and perhaps contradictory role of DNA methylation in the determination of recombination events in the model system *Drosophila melanogaster*. *D. melanogaster* possesses the DNA methyltransferase DNMT2 and a methyl binding domain protein, MBD2/3, which typically binds to methylated DNA and recruits chromatin remodeling complexes (Tweedie *et al.* 1999; Roder *et al.* 2000; Ballestar *et al.* 2001; Marhold *et al.* 2004a). Transcripts of *Dnmt2* were particularly enriched during early stages of embryonic development; expression in adult flies was limited to female ovaries, and there was no activity in male testes (Lyko *et al.* 2000), consistent with a role in recombination, which is female-specific in *Drosophila*. Furthermore, like MBD2 knockout mice, *Drosophila* null mutants of MBD2/3 were viable and fertile but revealed chromosome segregation defects (Lyko *et al.* 2006).

DNA methylation has experienced a controversial history in *Drosophila*, but has been reportedly detected at low levels, ranging from 0.21 to 1% across the *Drosophila* genus (Tweedie *et al.* 1999; Gowher *et al.* 2000; Lyko *et al.* 2003a; Marhold *et al.* 2004b; Salzberg *et al.* 2004; D’avila *et al.* 2010; Takayama *et al.* 2014). One of its functions in *Drosophila* was thought to be retrotransposon silencing and stabilization of repeats, similar to its role in vertebrates and plants (Salzberg *et al.* 2004; Phalke *et al.* 2009; Krauss and Reuter 2011). In a new study, researchers reveal a wholly unique DNA methylation pattern in *D. melanogaster*, finding DNA methylation to be very localized, strand asymmetrical, dynamic, concentrated in CA- and CT-rich five base pair motifs, and most likely involved in gene expression (Takayama *et al.* 2014). The role of DNMT2 in this recent study is nonintuitive; in its absence, DNA methylation remains, although with altered patterns (Takayama *et al.* 2014).

Therefore, to determine whether there is a detectable effect of DNA methylation and/or the DNA methyltransferase *Dnmt2* on the distribution and frequency of recombination rate in *Drosophila*, I assayed recombination at one heterochromatic region spanning the centromere of chromosome 3 and two euchromatic regions on the X chromosome in *Dnmt2* −/− and control (*Dnmt2* +/−) *D. melanogaster*. I did not detect any change in recombination rate or distribution in the absence of *Dnmt2* and conclude that other epigenetic factors are determining sites of recombination events in *Drosophila*.

## Materials and Methods

### Stocks and crossing scheme

**Figure 1:**
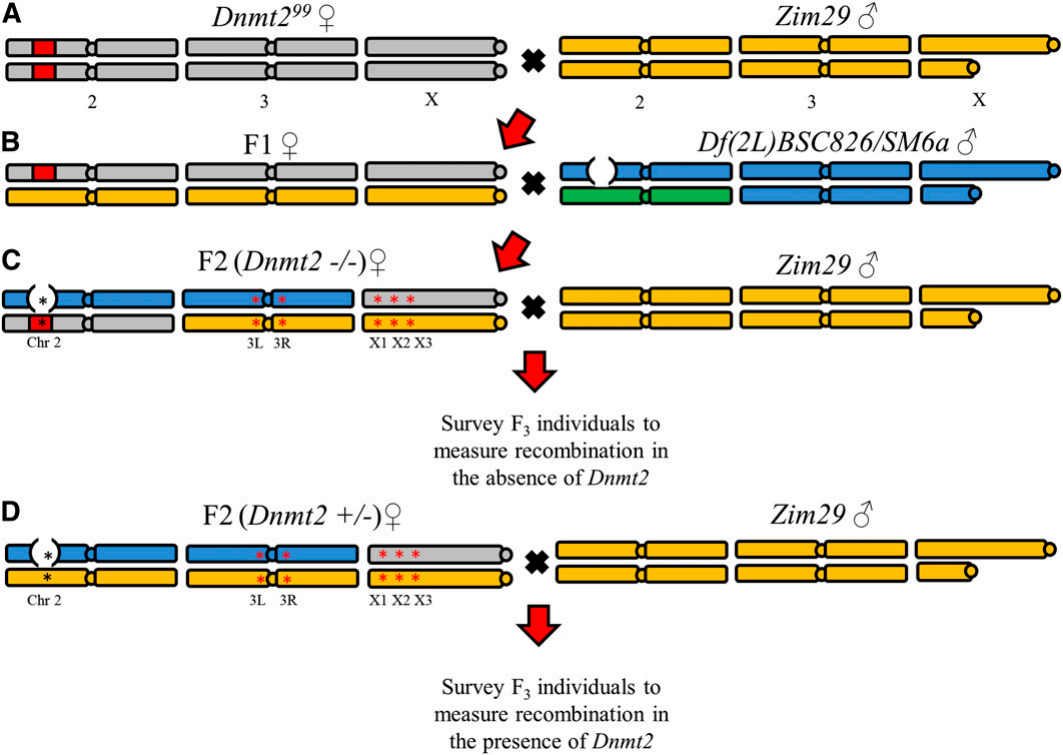
Crossing scheme. The crossing scheme follows the genotype of Chromosomes 2, 3, and X and consists of: (A) crossing a *D. melanogaster Dnmt2* p-element excision line *Dnmt2^99^* (gray, with p-element excision denoted in red) (Schaefer *et al.* 2010) to wild-type *D. melanogaster Zim29* (yellow) to generate variability to score recombination events. (B) F_1_ females were crossed to a *D. melanogaster* chromosome 2L deficiency line (blue, with deficiency denoted by an open circle) over a balancer (green), *Df(2L)BSC826/SM6a* (#27900 Bloomington Stock Center, Bloomington, IN). F_2_ females were collected, and females carrying the SM6a balancer were identified by the curly wing phenotype and discarded. (C) F_2_ females (*Dnmt2^99^/Df(2L)BSC826*) were crossed to wild-type males (*Zim29*), and experimental recombination was surveyed in the F_3_ progeny. (D) F_2_ females (*Df(2L)BSC826/Zim29*) were crossed to wild-type males (*Zim29*) and control recombination was surveyed in the F_3_ progeny. Markers used to assess genotype of F2 females and to assay recombination in F3 progeny are denoted in (C) and (D) as asterisks. More information about the markers is included in Table 1.

For all crosses, virgin flies were collected, separated by sex, and aged for 7 d. The crossing scheme (Figure 1) consisted of the following: (A) crossing a *D. melanogaster Dnmt2* p-element excision line *Dnmt2^99^*(Schaefer *et al.* 2010) to wild-type *D. melanogaster Zim29* to generate variability to score recombination events. (B) F_1_ females were crossed to a *D. melanogaster* chromosome 2L deficiency line over a balancer, *Df(2L)BSC826/SM6a* (#27900; Bloomington Stock Center, Bloomington, IN). F_2_ females were collected, and females carrying the SM6a balancer were identified by the curly wing phenotype and discarded. (C, D) The remaining F_2_ females (bearing 0−1 functional copies of *Dnmt2*) were crossed to wild-type males (*Zim29*) in single pair crosses, allowed to lay eggs, and subsequently genotyped after larvae appeared. Undesired genotypes (see section *Scoring recombination* for genotyping methods) were discarded, and F_3_ progeny were collected from remaining vials. Recombination was assayed in these individuals.

### Reverse transcription

To ensure that individuals of *Dnmt2^99^*/ *Df(2L)BSC826* genotype were *Dnmt2*−/−, reverse-transcription polymerase chain reaction (PCR) was completed. For each line (*Dnmt2^99^*, *Df(2L)BSC826/SM6a*, *Zim29*, *Dnmt2^99^*/ *Df(2L)BSC826*), I prepped RNA from approximately 25 pooled flies of varying life stages using the QIAGEN RNeasy kit, QIAGEN QIAShredder kit, and QIAGEN DNase kit. For each line, I used genomic DNA, H_2_O, and a negative control reverse transcription product as controls. For the reverse transcription reaction, the following recipe was used: 2 µL of 10× PCR buffer (15 mM MgCl_2_), 1.5 µL of 50mM MgCl_2_, 0.8 µL of 25 mM dNTPs, 1 µL of 50 µM experimental R primer, 1 µL of 50 µM control intron R primer, 0.5 µL of 40U/µL RNase inhibitor, 0.1 µL of 200U/µL MMLV reverse transcriptase, 9.1 µL of diethylpyrocarbonate H_2_O, and 4 µL of RNA mix + H_2_O to get to 500 ng of RNA. The reverse transcription program consisted of: 15 min at 42° followed by 5 min at 99°. Then, a PCR was completed with the following recipe: 2.5 µL of 10× PCR buffer (15 mM MgCl_2_), 1.5 µL of 2mM dNTPs, 1.25 µL, 10 µM experimental F primer, 1.25 µL of 10µM experimental R primer, 0.3 µL of DNA polymerase, 16.2 µL of H_2_O, and 2 µL of reverse transcription reaction product. The PCR program consisted of an initial denaturing step of 95° (60 sec), three touch-down cycles of 94° (30 sec)−56° (30 sec)−72° (45 sec) each, three touch-down cycles of 94° (30 sec)−53° (30 sec)−72° (45 sec) each, followed by 33 main cycles of 94° (30 sec)−50° (30 sec)−72° (45 sec) each. I used the *trade embargo* (TREM) gene as a control with the following primers: forward: CAGTAAGTGTGAATCCTGCTTGGTTTGC; reverse: GCATGTCCATAATGTGCTGATGGGATC. The primers used for flanking the *Dnmt2* intron were: forward: GGTCTTAGAACTATTTAGTGGCATTGGCG; reverse: TAATTGTGCGCATAAACCGCATTGGC.

### Scoring recombination

Flies were collected in 96-well plates and frozen at −20°. DNA was extracted following the protocol of Gloor and Engels (1992), using 49.5 μL of squish buffer [10 mM Tris-HCl (pH 8.2), 1 mM ethylenediaminetetraacetic acid, 25mM NaCl] + 0.5 μL of proteinase-K. A zirconium bead was placed in each well, and plates were then shaken using a QIAGEN TissueLyser II for 45 sec. The DNA preps were then incubated at 37° for 30 min and 95° for 2 min in a thermal cycler. The PCR recipe consisted of 0.5 μL of forward primer +M13 tag (CACGACGTTGTAAAACGAC added to 5′ end of forward primer), 0.5 μL of reverse primer, 0.4 μL of 700IRD or 800IRD-labeled M13 tag, 1.3 μL of MgCl2, 10× buffer, 1 μL of 2 mM dNTPs, and 0.2 μL of Taq polymerase in a 10-μL reaction volume. The same PCR program was used as described previously. Products were visualized on a polyacrylamide gel using a LICOR 4300.

To identify crosses with desired genotypes, F_2_ females were genotyped at three loci on the X chromosome (Table 1 and Figure 1, C and D) to ensure heterozygosity (*Df(2L)BSC826/Zim29*) across the region of interest, and at one locus on chromosome 2L (Table 1 and Figure 1, C and D) to identify if the genotype was *Dnmt2^99^/ Df(2L)BSC826* (*Dnmt2*−/−, experimental) or *Df(2L)BSC826/Zim29* (*Dnmt2* +/−, control). Vials from parents of the desired genotypes were kept, and the F_3_ progeny were collected; all other vials and their progeny were discarded.

**Table 1:** Genetic markers used to score recombination

Marker Name	Primer Name	Physical Distance/Genetic Distance Between Markers	Sequence (F/R)
Marker X1	DMELX_494471F	Marker X1-Marker X2: 2.75 Mb/5 cM	CGAGCGCTGTCTATTGCGTTC
	DMELX_494621R		TCATTTCAATTCCGATTTGGAGTCGGC
Marker X2	DMELX_3240050F	Marker X2-Marker X3: 2.68 Mb/10 cM	GGAAACAGTGTTATTGCCTACACATGGAAC
	DMELX_3240200R		CTTGGCCAAGTTGCACATGAGATAC
Marker X3	DMELX_5922532F	Marker X1-X3: 5.43 Mb/15 cM	GGATCGTTGCAGATCGGATAGAACTC
	DMELX_5922673R		CCGTCTCAAATTGATGGACGCCTAT
Marker Chr2	DMEL2L_12024260F	NA	CGTCACATTCCATTGAACGACTTTCGG
	DMEL2L_12024434R		CAAAACTGGCTCCAAACGTCCGTG
Marker 3L*^a^*	DMEL3L_16327010F	Marker 3L-Marker 3R: 12.75 Mb/4 cM	GATTCAACTGACGTCACCAGATGAGC
	DMEL3L_16328059R		CGCCTCTTTCGAATTGCATCACTGAG
Marker 3R	DMEL3R_4531346F		CACCCTCGAAAAAAGTTGCCAACGT
	DMEL3R_4531276R		CAAAGTGTATCTTCATCGCCGACTCAC

NA, not available.

a Used in conjunction with the restriction enzyme *Bso*BI. See the section *Materials and Methods*.

Recombination was scored in F_3_ progeny by genotyping at the same three markers on the X chromosome. The markers on the X chromosome delineate two regions, one of lower recombination (~1.8 cM/Mb) and one of higher recombination (~3.7 cM/Mb). A recombinant was called when an individual fly’s genotype changed from heterozygous to homozygous or vice versa for females, and when the fly’s genotype changed between the possible allele combinations for the males. In total, 1536 F_3_ control progeny and 1177 F_3_ experimental progeny were scored for the euchromatic regions.

An additional heterochromatic region was added later, and a subset of the same pool of F_2_ females were selected based on exhibiting heterozygosity in this additional region of interest at two markers spanning the centromere of Chromosome 3 (Table 1 and Figure 1, C and D). For the Chromosome 3L marker, the *Bso*BI enzyme (New England BioLabs) was used with the following conditions: 10 μL of PCR product, 1.2 μL of CutSmart Enzyme buffer, 0.6 μL of *Bso*BI enzyme, and 8.2 μL of ddH_2_O, incubated at 37° for 60 min, 80° for 20 min, and 10° for 1 min. The digests were run on 1% agarose gels at 160V for 45 min. Recombination was scored in F_3_ progeny with the appropriate parental genotypes, a subset of the total F_3_ progeny described previously. This resulted in a more modest sample size of 200 F_3_ control progeny and 152 F_3_ experimental progeny scored for the heterochromatic region.

### Statistics

Recombination fractions between experimental and control individuals were compared using an unpaired *t*-test (GraphPad Software, Inc. La Jolla, CA). A power analysis was completed using the “pwr” package in R [Statistic: (Cohen 1988) R package: Stephane Champely].

## Results

### Recombination across a euchromatic region

To identify an effect of the DNA methyltransferase gene *Dnmt2* on meiotic recombination, I created a variable *Dnmt2* null fly stock (confirmed with reverse transcription) and identified recombinants in two adjacent euchromatin regions on the X chromosome. Region 1 spans assembly positions 494,471 to 3,240,200 bp (Adams *et al.* 2000) and represents a region of relatively low recombination (2.75 Mb, approximately 5 cM), whereas Region 2 spans assembly positions 3,240,200 to 5,922,673 bp and represents a region of relatively high recombination (2.68 Mb, 10 cM). This delineation was used to account for possible large-scale changes in the distribution of recombination events in euchromatin in the absence of *Dnmt2*.

**Figure 2:**
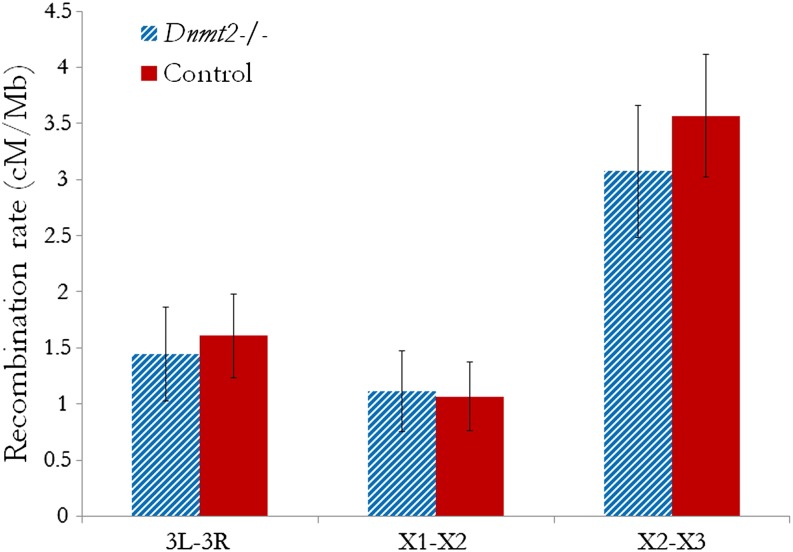
No difference in recombination rate between *Dnmt2*−/− and *Dnmt2*+/− individuals. Recombination rates (cM/Mb) are shown for *Dnmt2*−/− individuals (blue column) and *Dnmt2*+/− individuals (red) across three intervals: 3L-3R, which spans the heterochromatic centromere of chromosome 3 (*P* = 0.79; Control: 1.61 cM/Mb; Experimental: 1.44 cM/Mb); X1-X2, a euchromatic region of the X chromosome with low recombination (*P* = 0.86; Control: 1.07 cM/Mb; Experimental: 1.11 cM/Mb); and X2-X3, a euchromatic region of the X chromosome with high recombination (*P* = 0.84; Control: 3.57 cM/Mb; Experimental: 3.07 cM/Mb). The error bars reflect 95% confidence intervals. There is no significant difference between experimental and control individuals for any interval surveyed.

In total, 1536 *Dnmt2*+/− (control) individuals and 1177 *Dnmt2*−/− individuals were scored. In Region 1, I identified no significant difference between *Dnmt2* −/− and control individuals (*P* = 0.86; Control: 3.02% recombinant, 1.07 cM/Mb; Experimental: 3.16% recombinant, 1.11 cM/Mb; Figure 2). Results were similar for Region 2 (*P* = 0.84; Control: 10.58% recombinant, 3.57 cM/Mb; Experimental: 8.98% recombinant, 3.07 cM/Mb; Figure 2). A power analysis showed with these sample sizes, I could detect an effect size of d = 0.1 (power = 0.8, significance level 0.05).

### Recombination across a heterochromatic region

Upon finding no significant difference in euchromatic recombination rates, I sampled recombination rates for a subset of the aforementioned individuals across the heterochromatic centromere of Chromosome 3. This was done to account for possible changes in the distribution of recombination rate across a heterochromatic region in the absence of *Dnmt2*, as seen in Arabidopsis (Melamed-Bessudo and Levy 2012; Mirouze *et al.* 2012; Yelina *et al.* 2012). I scored 200 *Dnmt2* +/− individuals and 152 *Dnmt2* −/− individuals at markers at assembly positions 3L: 16,327,010 and 3R: 4,531,276, and once again found no significant difference in recombination rates (*P* = 0.79; Control: 20.5% recombinant, 1.61 cM/Mb; Experimental: 18.4% recombinant, 1.44 cM/Mb; Figure 2). With this diminished sample size, the power to detect small differences in recombination rate is naturally decreased; (power = 0.8, significance level 0.05; N= 200, d = 0.28; N = 152, d = 0.32), although the results are consistent with the increased sample size results from the euchromatic region. I therefore conclude that the DNA methyltransferase DNMT2 has no detectable effect on recombination in *D. melanogaster*.

## Discussion

Although DNA methylation influences the recombination landscape in fungus *A. immersus* and Arabidopsis, I detected no effect of knocking out the DNA methyltransferase *Dnmt2* on recombination in *Drosophila*. The crossover analyses were limited to two genomic regions, but these regions captured both low- and high-recombination areas of euchromatin, and the centromere of chromosome 3, a highly repetitive heterochromatic region. It remains possible that DNA methylation exhibits intergenerational epigenetic inheritance, as the *Dnmt2−/−* females were created using a heterozygous (*Dnmt2* +/−) mother (Figure 1), but this seems unlikely. Organisms typically undergo one or more rounds of epigenetic reprogramming, in which epigenetic signatures are erased and reprogrammed in the germline and in the early embryo. The failure of this process results in epigenetic inheritance, and although there is evidence of this occurring, especially in plants, it is not thought to be a widespread phenomenon (Heard and Martienssen 2014). As such, DNMT2 appears to have no major effect on rates of recombination in specific regions of the genome, and DNA methylation more generally also may not affect recombination in *Drosophila*.

The presence of DNA methylation in *Drosophila* and other *Dnmt2*-only systems is a long-debated issue (Schaefer and Lyko 2010; Raddatz *et al.* 2013). Although the protein DNMT2 shows strong sequence and structural conservation to established methyltransferases, the enzymatic activity was found to be much weaker (Okano *et al.* 1998; Dong *et al.* 2001). Various studies reporting DNA methylation in *Drosophila* (Tweedie *et al.* 1999; Gowher *et al.* 2000; Lyko *et al.* 2000; Lyko 2001; Lyko *et al.* 2003a,b; Marhold *et al.* 2004a,b; Salzberg *et al.* 2004; Phalke *et al.* 2009; Krauss and Reuter 2011) may have been confounded by contaminations from other organisms, detection limits, low antibody specificity, and/or false positives (Zemach *et al.* 2010; Raddatz *et al.* 2013). More advanced bisulfite sequencing with thorough controls even questioned the existence of DNA methylation in *Drosophila* (Raddatz *et al.* 2013). Alternatively, a new study that enriched methylated DNA through immunoprecipitation before bisulfite conversion has revealed a unique and dynamic genomic methylation pattern, suggesting that previous studies (Zemach *et al.* 2010; Raddatz *et al.* 2013) lacked sufficient coverage to detect methylation (Takayama *et al.* 2014). This most recent study presents strong evidence for an unidentified *de novo* methyltransferase in *Drosophila*, showing that genomic methylation persists in the absence of DNMT2 (although with altered patterns of DNA methylation) (Takayama *et al.* 2014).

The finding that there is no effect of the gene *Dnmt2* on meiotic recombination supports this recent data and points to other epigenetic mechanisms directing recombination in *Drosophila*. Indeed, there is some evidence that histone modifications in *Drosophila* may mimic the role of DNA methylation in transcriptional processes in other invertebrates (Cedar and Bergman 2009; Chodavarapu *et al.* 2010; Nanty *et al.* 2011; Hunt *et al.* 2013). Additionally, although DNA methylation was reportedly involved in specific transposons in *Drosophila* (Phalke *et al.* 2009), the small RNA Piwi-piRNA pathway is known to be the main genome defense system against repetitive elements in the germline (Aravin *et al.* 2007; Brennecke *et al.* 2007; Blumenstiel 2011).

Clearly, *Dnmt2* and *Drosophila* DNA methylation research has experienced a tumultuous 20 years, although new research appears to settle the question of the existence of DNA methylation in *Drosophila* (Takayama *et al.* 2014). Whether DNA methylation influences the recombination landscape in organisms besides the fungus *A. immersus* and Arabidopsis is one question that remains to be elucidated, but I conclude based on the available results that DNMT2-dependent methylation has no detectable role in *Drosophila* recombination.
